# The C.O.S. and the Preparation of Charitable Accounts

**Published:** 1890-04-05

**Authors:** 


### Passing Topics.

### THE C. O. S. AND THE PREPARATION OF CHARITABLE ACCOUNTS.

We are among those—none too many—who think that the Charity Organization Society deserves well of the Nation, if only because of the example it sets in the patient processes of its complex and somewhat inscrutable purposes ; in its never-ceasing concern for the improvement of undertakings and individuals who are well content to remain as they are ; and the sublime insensibility to ingratitude which marks the true philanthropist.

We can well believe that had the Society flourished at an earlier period of the world's history—in the days of the Georges, for instance—it might have attracted those grosser attentions with which the caricaturists of a vicious age were always ready to embarrass unconscious virtue ; but the refining influences of the later times ensure for it the chaste admiration due to high art. That the workers of the C. O. S. are true artists is evident when we reflect how entirely they abandon themselves to the task of perfecting their handiwork, undeterred by considerations of its utility, and indifferent to the certainty that it will bring them neither honour nor reward.

There are few societies, no matter how robust their faith in their own panaceas, which could have brought to bear a year's thought and labour of a sub-committee of fourteen persons, assuredly not idle men, upon the production of the "Report on the Preparation and Audit of the Accounts of of Charitable Institutions," which was recently considered and adopted by the council. Issued from the office of the Queen's Printers, and bearing not a little resemblance to an Act of Parliament, this portentous document of twenty-two pages embodies some of the abstrusest and yet most unquestionable theories concerning accounts we have been privileged to meet with. It is no fault of the Sub-Committee that their chief proposals are not altogether new, for truth, we know, is eternal; but they are in no wise less valuable, or, so far as the committee is concerned, less original on that account. What, for instance, can be more important and noteworthy than the series of recommendations with which the report ends, embracing as it does the following items amongst others: That the cash-book should be kept upon a columnar method; that the books should be balanced and the statement for audit prepared prior to the visit of the auditors ; and that the bankers' pass-books should be made up to the end of the period under review.

It says something for the thoroughness of the sub-committee's work that these elementary matters have not been allowed to escape them, and it behoves the managers of charitable institutions to take heed lest, after this clear exposition of their duties, they allow a non-columnar cash-book upon the premises, permit an auditor to enter upon an examination of unbalanced accounts, or expect him to take the entries in the pass book on trust. If they do they will know what to expect at the hands of their guide, philosopher, and—censor. Nor will a plea of ignorance of the law, the law of the C.O.S., avail, even were it possible, after the publication of the several "suggested forms of accounts" with which the later pages of this report are charged. These are designed to meet the requirements of all—from "small institutions without property and trading or indebtedness"—happy, but somewhat invertebrate, organizations these, one would suppose—to "large institutions having current liabilities at the end of the period." Although it may seem there is an excess of elaboration about this akin to the provision of two entrances to the fowl house, the larger one for the full-grown fowls, and the smaller for the chickens, it is better, doubtless, to have too much rather than too little of a good thing, and we all know how difficult it is to hit the exact mean.

Were we disposed to cavil, we might regret that a document already so comprehensive was not rendered absolutely exhaustive. For example, would it not have been well to require that entries should be made in permanent ink, and to have entered a protest against blots and dog's ears? The expression of a hope by the sub-committee that "the results of its labours may be found not only in the acceptance (by the council) of the methods and forms indicated, but by their adoption generally by charitable institutions" is too modest. We are assured that some of the committee's recommendations were anticipated by past generations, and have stood the test of time, while one critic remarks that he has made use of a certain suggested form for twenty years. How can the eleven months' labour of this devoted body be better justified than by testimony like this? Agricola.

